# Effects of bile acids and gut microbiota interactions on neurological and psychiatric disorders

**DOI:** 10.1016/j.gendis.2026.102069

**Published:** 2026-02-06

**Authors:** Xiaoqi Pei, Xiaoli Wang, Hongji Lu, Jialin Wu, Lu Xu, Xiaoyin Chen, Li Deng, Huachong Xu

**Affiliations:** a School of Traditional Chinese Medicine, Jinan University, Guangzhou, Guangdong 510632, China; b Key Laboratory of CNS Regeneration (Jinan University), Ministry of Education, Guangzhou, Guangdong 510632, China; c Guangdong Provincial Key Laboratory of Traditional Chinese Medicine Informatization, Jinan University, Guangzhou, Guangdong 510632, China

**Keywords:** Bile acids, Gut–brain axis, Gut microbes, Neuroimmunity, Neurological disorders

## Abstract

The gut–brain axis, as a bidirectional communication network connecting the gut microbiota to the central nervous system, plays a key role in a variety of neurological and psychiatric disease processes. In recent years, researchers have discovered that bile acids (BAs) are important signaling molecules that extend beyond their traditional role in lipid digestion and absorption. They are now known to play a significant role in regulating metabolism, immunity, and nervous system functions. Notably, gut microbes dynamically regulate BA profile through deconjugation, dehydroxylation, and other biotransformations, building a complex regulatory network of microbial–host co-metabolism, which in turn modulates gut–brain axis function through multiple receptors. Specifically, BAs precisely regulate neuroimmune homeostasis, dynamic balance of the blood–brain barrier, and neurotransmitter metabolism through the activation of membrane and nuclear receptors, such as farnesoid X receptor (FXR), takeda G protein-coupled receptor 5 (TGR5), pregnane X receptor (PXR), vitamin D receptor (VDR), and sphingosine-1-phosphate receptor 2 (S1PR2), a mechanism that provides a new perspective for elucidating the pathogenesis of neurological and psychiatric diseases. In this review, we systematically elucidate the microbial regulatory mechanisms of BA metabolism, focusing on the pathways through which BA–gut microbiota interactions affect neurological and psychiatric disorders via the neuroimmune pathway. By integrating the latest research evidence, we systematically sort out the key signaling pathways mediated by BA receptors and their neuroimmune regulatory networks, and provide a theoretical framework for the development of targeted intervention strategies for psychiatric disorders based on the regulation of the gut–brain axis.

## Introduction

In recent years, the gut–brain axis, as a crucial bidirectional signaling network connecting the gut and the central nervous system (CNS), has become a hotspot in neuropsychiatric disease research. The gut microbiota, serving as a key hub of the gut–brain axis, can regulate brain function and behavior via metabolites, immune signals, and neurotransmitters. Substantial evidence indicates that gut microbiota dysbiosis is closely associated with various nervous system disorders, and microbe-derived metabolites play a critical signaling role in this process. Among these metabolites, bile acids (BAs) have garnered increasing attention due to their multiple functions in metabolism, immunity, and neuroregulation.^1^

Initially, research on BAs did not focus on the nervous system but rather stemmed from the exploration of their metabolic transformation pathways. In the mid-to-late 20th century, chemical analysis methods detected secondary BAs in human feces with compositions different from the primary BAs synthesized by the liver. This discovery suggested that gut microbiota might be involved in the biotransformation of BAs.^2^, ^3^, ^4^ Subsequently, during the 1970s–1980s, the research emphasis shifted to elucidating key metabolic steps, including the deconjugation reaction mediated by bile salt hydrolase (BSH) and the 7α-dehydroxylation pathway carried out by anaerobic bacterial communities. The mechanistic insights gained during this phase transitioned the enterohepatic circulation from a conceptual framework to a biochemical model verifiable through specific metabolic reactions.^5^, ^6^, ^7^, ^8^ Entering the 21st century, breakthroughs in high-throughput sequencing, high-resolution mass spectrometry, artificial intelligence, and multi-omics integration enabled researchers to systematically identify key microbial communities, functional gene clusters, and the structural diversity of metabolites involved in BA transformation.^9^, ^10^, ^11^, ^12^ Concurrently, the discovery of nuclear receptors and membrane receptors redefined BAs from traditional lipid-digesting molecules to important signaling molecules capable of regulating host metabolic homeostasis, immune responses, and inflammatory processes through receptor-mediated pathways.^13^, ^14^, ^15^, ^16^ With the expansion of research horizons, the functional associations of BAs have extended from the gut–liver axis to the gut–liver–brain axis. Recent studies have shown that gut microbiota-regulated BA profile can participate in modulating the release of inflammatory factors, blood–brain barrier (BBB) permeability, and neurotransmitter metabolism via the circulatory system, thereby influencing CNS functions and contributing to processes such as emotion, cognition, and neuroinflammation.^17^^,^^18^ These advances not only underscore the important signaling role of BAs in the nervous system but also suggest their potential pathological significance in the regulation of psychiatric disorders.

In our previous studies, we have shown that high-humidity and high-temperature environments can alter gut microbiota and host immune status, thereby exacerbating pulmonary,^19^, ^20^, ^21^, ^22^, ^23^ intestinal,^24^ and psychiatric disorders.^25^ Chinese herbal medicines can restore the structure of gut microbiota and play important roles in host metabolism, immunity, and anti-inflammatory responses. Therefore, we have conducted a series of studies focusing on how Chinese herbal medicines and their active components treat diseases by regulating gut microorganisms. For instance, high-humidity and high-temperature environments can impair intestinal barrier function, disrupt tryptophan metabolism, and dysregulate the aryl hydrocarbon receptor/IL-22 signaling pathway. Key genes such as nucleotide-binding oligomerization domain 2 (NOD2), nuclear factor κB (NF-κB), and polymeric immunoglobulin receptor (pIgR) are up-regulated, ultimately leading to aggravated local inflammation, increased intestinal permeability, and prolonged disease course.^20^ *Gegen Qinlian Decoction* has been shown to modulate systemic immunity through remodeling gut microbiota, thereby protecting mice from influenza viral pneumonia.^21^ Another example is *Poria cocos* polysaccharide, which may improve antibiotic-associated diarrhea in mice by modulating gut microbiota, thereby influencing intestinal mucosal barrier function, host immune responses, and metabolic functions.^24^ In a 2024 study on anxiety disorders, we further show that gut dysbiosis induced by high-humidity and high-temperature environments, characterized by a reduction in *Lactobacillus*, disrupts BA metabolism, elevates serum levels of secondary BAs such as lithocholic acid (LCA), and promotes the progression of anxiety by driving neuroinflammation.^25^

Based on the above background, this article focuses on BA metabolism and gut microbial regulatory mechanisms, and systematically reviews the effects of BA–gut microbiota interactions on neuroimmune function and related diseases. With a focus on BA receptor-related signaling pathways, it examines the mechanisms of gut dysbiosis and abnormal BA metabolism on neurological and psychiatric disorders. It aims to provide new ideas for the research on the pathological mechanisms and intervention strategies of the gut–brain axis.

## Overview of BA metabolism

The core function of BAs as amphiphilic molecules is to facilitate lipid digestion and absorption. However, recent studies have revealed that BAs can also play significant roles in metabolic regulation, immune regulation, and the maintenance of gut microbial homeostasis through diverse molecular forms and receptor-mediated signaling pathways.

BAs can be categorized by structure into unconjugated bile acids (UCBAs) and conjugated bile acids (CBAs), and by origin into primary BAs and secondary BAs.^26^ BAs synthesized in hepatocytes from cholesterol through a series of enzymatic reactions are called primary BAs.^27^ Primary BAs conjugate with taurine or glycine, forming CBAs, which are stored in the gallbladder as bile salts.^28^^,^^29^ During feeding, primary CBAs enter the intestine and undergo various modifications by gut microbiota.^30^ Deconjugation releases the originally bound glycine or taurine, converting the BAs into free BAs. Free BAs undergo removal of the 7α-hydroxy group to form more hydrophobic secondary BAs. Gut microbiota can also mediate the conjugation of bile acids, producing many microbe-conjugated bile acids.^31^ These microbe-conjugated bile acids can influence host metabolism, such as by modulating the activity of BA receptors, thereby affecting energy metabolism, inflammatory responses, and immune regulation. Most BAs are reabsorbed before reaching the terminal ileum and return to the liver via the hepatic portal vein, a process known as the enterohepatic circulation.^32^^,^^33^ Free BAs have low polarity and high lipid solubility, enabling them to directly cross the lipid bilayer of intestinal epithelial cells via passive diffusion for absorption, without energy consumption or reliance on specific transporters. CBAs have higher polarity and water solubility, thus requiring specific transporter proteins, such as the apical sodium-dependent bile acid transporter (ASBT), for efficient absorption. The enterohepatic circulation of BAs allows for their multiple reuses, reduces cholesterol consumption, and maintains the dynamic balance of BAs in the body (Fig. 1).^34^^,^^35^

**Figure 1:**
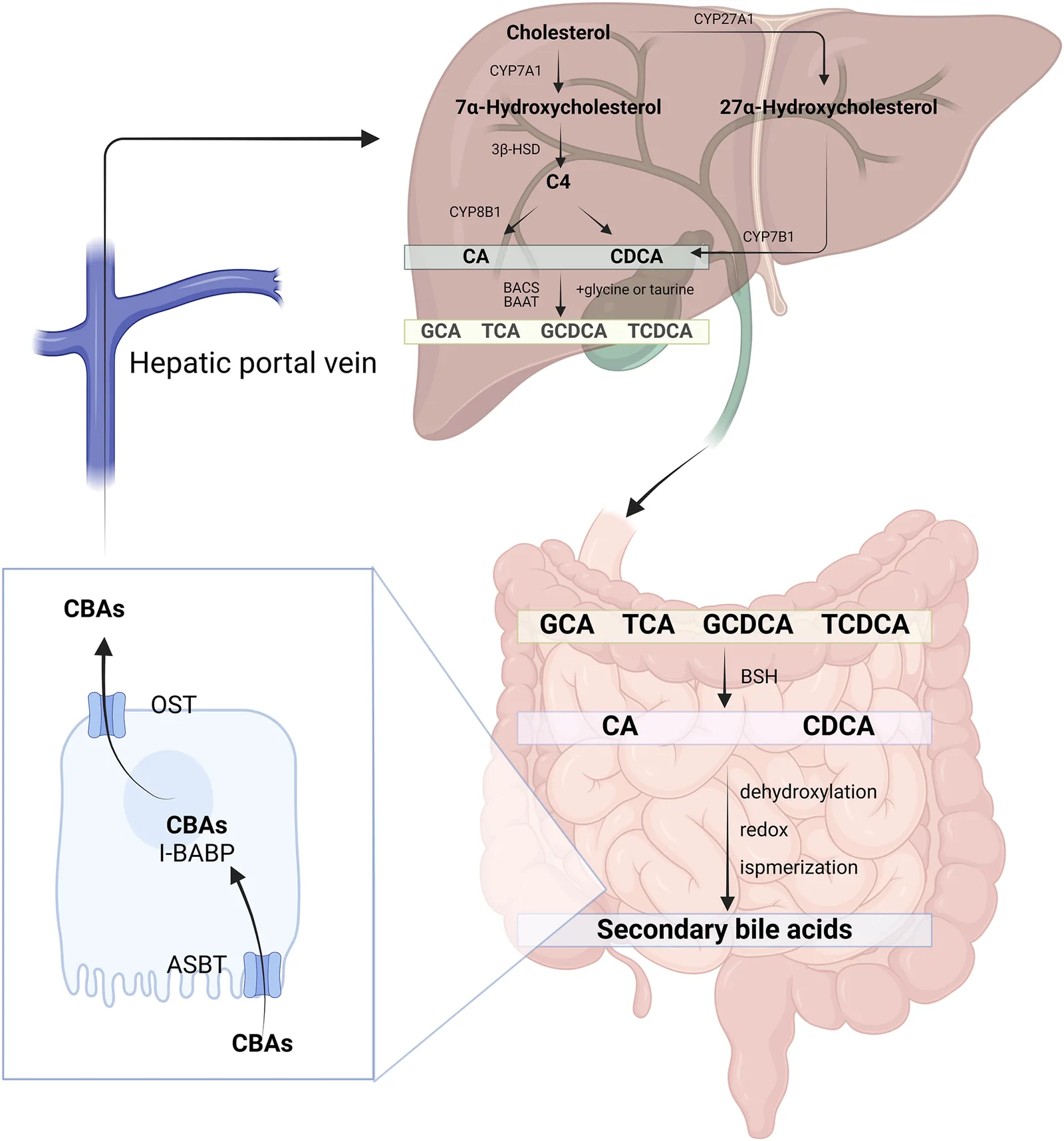
BA metabolism and enterohepatic circulation. Primary BAs are synthesized mainly through two pathways: the classical pathway and the alternative pathway. The classical pathway is initiated by CYP7A1, which catalyzes the conversion of cholesterol to 7α-hydroxycholesterol. This product is subsequently converted to C4 by 3β-HSD. CYP8B1 then catalyzes the transformation of C4 into 7α,12α-dihydroxy-4-cholesten-3-one, ultimately leading to the synthesis of CA. In the absence of CYP8B1 activity, CDCA is generated instead. The alternative pathway involves CYP27A1 hydroxylating the 27th position of cholesterol to form 27-hydroxycholesterol, which is then catalyzed by CYP7B1 to produce CDCA.BACS and BAAT catalyze the conjugation of primary BAs with taurine or glycine to form CBAs. In the terminal ileum, CBAs are taken up by ASBT and intracellularly transported by I-BABP, then excreted into the portal vein by the basolateral OST, and reabsorbed into the liver. BA, bile acid; CYP7A1, cholesterol 7α-hydroxylase; CYP27A1, sterol 27-hydroxylase; CYP7B1, oxysterol 7α-hydroxylase; CYP8B1, sterol 12alpha-hydroxylase; 3β-HSD, 3β-hydroxysteroid dehydrogenase; BACS, bile acid coenzyme A synthase; BAAT, bile acid-CoA: amino acid N-acyltransferase; BSH, bile salt hydrolase; ABST, sodium-dependent bile acid transporter protein; I-BABP, ileal bile acid binding protein; OST, organic solute transport protein; C4, 7α-hydroxy-4-cholesten-3-one; CA, cholic acid; CDCA, chenodeoxycholic acid; GCA, glycochenocholic acid; TCA, taurocholic acid; GCDCA, glycochenodeoxycholic acid; TCDCA, taurochenodeoxycholic acid; CBAs, conjugated bile acids.

BA receptors are a type of receptor protein that can recognize and bind to BAs, and they mediate the biological effects of BAs. In addition to playing a role in lipid digestion and absorption, BAs also act as signaling molecules, participating in the regulation of multiple physiological processes, including metabolism, immunity, inflammation, and neurological function by activating these receptors. BA receptors are primarily categorized into two main classes: nuclear receptors and membrane receptors. The details are provided in Table 1.

**Table 1:** BA receptors.

Type	Receptor	BA ligands	Main function
Nuclear receptor	FXR	Various BAs (CDCA > DCA > LCA > CA)^181^	Anti-inflammation, immune regulation, and tissue protection^36^^,^^78^, ^79^, ^80^, ^81^^,^^83^
	PXR	LCA and its derivatives^37^	Liver detoxification, drug metabolism, and neuroprotection^86^, ^87^, ^88^, ^89^
	VDR	LCA and its derivatives^39^^,^^40^	Regulates calcium and phosphorus metabolism and bone health; BA metabolism and intestinal immunity^90^, ^91^, ^92^
Membrane receptor	TGR5	Various BAs^97^	Inhibits inflammatory response and oxidative stress, and participates in neuroprotection^102^
	S1PR2	Conjugated BAs^47^^,^^182^	Regulates BBB, vascular function, and neuroinflammation^110^

Note: This table describes the main functions of BA receptors. BA, bile acid; CDCA, chenodeoxycholic acid; LCA, lithocholic acid; DCA, deoxycholic acid; CA, cholic acid.

The farnesoid X receptor (FXR) is a member of the nuclear receptor superfamily, belonging to subfamily 1, group H, and member 4. Therefore, it can be called NR1H4. It is encoded by the nuclear receptor gene NR1H4 in humans and is widely expressed throughout the body.^14^ BAs are the natural ligands of FXR, so FXR is also called the bile acid receptor (BAR).^13^ FXR exerts anti-inflammatory, immunomodulatory, and tissue-protective effects through multiple pathways, such as regulating BA metabolism, maintaining intestinal homeostasis, inhibiting inflammatory responses and regulating glycolipid metabolism, influencing neuroimmunity, and participating in tumor prevention.^36^

The pregnane X receptor (PXR), also known as nuclear receptor subfamily 1, group I, member 2 (NR1I2), belongs to the nuclear receptor superfamily. PXR is highly expressed in the liver and small intestine, as well as in the kidney, stomach, skin, and BBB. LCA and its oxidized form, 3-ketolithocholic acid (3-keto-LCA), have a strong activating effect on PXR.^37^ PXR was originally recognized as an exogenous substance receptor that regulates metabolic enzymes and transport proteins and plays a role in detoxification and drug metabolism in the liver. However, further studies have shown that PXR is also a receptor for endogenous substances that are equally important in metabolism and homeostasis.^38^

The vitamin D receptor (VDR) is a nuclear receptor that was initially discovered as a receptor for 1 alpha, 25-dihydroxyvitamin D_3_ (1,25(OH)_2_D_3_), regulating calcium and phosphorus metabolism and bone health. It is expressed in tissues involved in calcium and phosphate metabolism and in immune cells. VDR can also recognize certain BAs, especially secondary BA LCA, and plays an important role in BA metabolism and intestinal immunity.^39^^,^^40^

The Takeda G protein-coupled receptor 5 (TGR5), also known as G protein-coupled bile acid receptor 1 (GPBAR1),^16^ is a G protein-coupled receptor activated by BAs and plays an important role in neuroimmune regulation. Its expression in the CNS (such as microglia, astrocytes, and some neurons) and peripheral immune cells participates in neuroprotection by inhibiting inflammatory responses and oxidative stress.^41^

Sphingosine-1-phosphate receptor 2 (S1PR2) is a G protein-coupled receptor and a member of the S1PR family. It can be activated by specific CBAs. Its activation in microglia can promote neuroinflammatory responses, affecting the integrity of the BBB and the stability of the neuronal microenvironment.^42^, ^43^, ^44^ It is noteworthy that S1PR was initially known as endothelial differentiation genes (EDG), with five subtypes: EDG1, EDG5, EDG3, EDG6, and EDG8, now referred to as S1PR1–5. Among these, S1PR1 primarily promotes cell migration and vascular barrier stability; S1PR3 regulates vascular smooth muscle contraction and cardiovascular function; S1PR4 mediates immune regulation in immune cells; S1PR5 promotes myelination and neuroprotection in oligodendrocytes; whereas the function of S1PR2 contrasts with S1PR1, as its activation typically inhibits cell migration, increases endothelial permeability, and promotes inflammatory responses.^45^, ^46^, ^47^ Current literature reports indicate that S1PR2 can be activated by specific CBAs, while no such interactions have been reported for other members.

In addition to the aforementioned direct receptors for BAs, BA metabolism is also indirectly regulated by various receptors. For example, liver X receptor (LXR) can be activated by oxysterols, and its activation up-regulates the expression of the *CYP7A1* gene. CYP7A1 is the rate-limiting enzyme in the classical BA synthesis pathway, and its activity directly determines the amount of cholesterol converted into BAs.^48^, ^49^, ^50^ Peroxisome proliferator-activated receptor (PPAR) is activated by natural fatty acids. It coordinates the balance between overall energy metabolism and BA homeostasis by regulating fatty acid β-oxidation and the transcription of BA transporter proteins.^51^ Although these receptors do not directly sense BAs, they influence BA production at its metabolic source by regulating enzymes and transporters involved in cholesterol, lipid, or BA metabolism, thereby providing substrates and shaping the metabolic environment for BA synthesis.

## Regulation of BAs by gut microbes

### Mechanisms of gut microbiota involvement in BA deconjugation and transformation

In the complex physiological system of the human body, the gut microbiota plays an indispensable role, especially in the metabolic process of BAs. Primary BAs are transformed into various secondary BAs in the intestinal tract through the action of intestinal microorganisms, greatly enriching the BA profile and biological activity.^29^ Gut microbes convert BAs by four major pathways: deconjugation, 7α-dehydroxylation, redox, and isomerization. Recent studies have included recombination products of BAs as a fifth microbial modification, including the formation of BA-24-amidates with amino acids or polyamines at the C-24 position and BA-3-O-acylates with fatty acids or organic acids at the C-3 position.^10^^,^^52^ Through these pathways, BAs have been endowed with more diverse biological functions and play an important role in the regulation of the liver–gut–brain axis.

### Key gut microbiota and functional enzymes

The gut microbiota, through its diverse enzyme systems, performs a series of complex biotransformations on BAs, influencing the structure and function of BAs and forming a complex network of microbial–BA interactions. This network not only maintains gut microecological homeostasis but also regulates host metabolism, immunity, and neurological functions through BAs as signaling molecules.

Deconjugation via BSH is a critical step for further microbial modification of BAs in the intestinal environment. The deconjugated UCBAs are more susceptible to further conversion, and deconjugation is a prerequisite for subsequent 7α-dehydroxylation and redox reactions. BSH is widely present in a broad range of enteric bacterial genera, including gram-positive bacteria such as *Clostridium*,^53^ *Lactobacillus*,^54^ *Bifidobacterium*,^55^ and *Enterococcus*,^56^ as well as in *Bacteroides*^57^ and other gram-negative bacteria, all of which exhibited BSH activity.^29^ BSH catalyzes the hydrolysis of CBAs to UCBAs, reducing their water solubility and facilitating subsequent microbial transformation processes. This process not only affects the intestinal luminal concentration and physicochemical properties of BAs but also regulates the inhibitory selective effects of BAs on the microecological environment, thus indirectly shaping the composition and diversity of the gut microbiota.

The 7α-dehydroxylation is one of the core steps for gut microbiota to transform BAs. Some members of the genus *Clostridium*, including *Clostridium scindens* (*C. scindens*), *Clostridium hiranonis* (*C. hiranonis*), *Clostridium hylemonae* (*C. hylemonae*) (cluster XIVa), and *Clostridium sordellii* (*C. sordellii*) (cluster XI).^58^^,^^59^ The *bai* (bile acid-inducible) gene cluster encodes 7 alpha-dehydroxylase (7α-dehydroxylase).^60^ This enzyme catalyzes the 7α-dehydroxylation of primary BAs (*e.g.*, CDCA, CA) to form secondary BAs (*e.g.*, LCA, DCA). These secondary BAs have stronger biofilm penetration ability and higher receptor affinity, and can significantly affect the functional status of local and distal intestinal organs, such as the CNS.

In addition, the gut microbiota can also catalyze the hydroxyl redox and isomerization reactions of BAs, converting the hydroxyl groups from the α direction to the β direction, thereby generating BAs with stronger hydrophilicity and lower toxicity, further enriching the types of BAs and their biological effects. For example, some gut bacterial strains catalyze the hydroxyl oxidation and stereoconformational transition of BAs via 3 alpha-Hydroxysteroid dehydrogenase (3α-HSDH), 3 beta-Hydroxysteroid dehydrogenase (3β-HSDH), 7 alpha-Hydroxysteroid dehydrogenase (7α-HSDH), 7 beta-Hydroxysteroid dehydrogenase (7β-HSDH), 12 alpha-Hydroxysteroid dehydrogenase (12α-HSDH) and 12 beta-Hydroxysteroid dehydrogenase (12β-HSDH), generating a wide range of epi-BA and oxo-BA derivatives, which are metabolites that have been demonstrated to be important in the regulation of neuroinflammatory, gut-brain signaling (Fig. 2; Table 2).^61^^,^^62^

**Figure 2:**
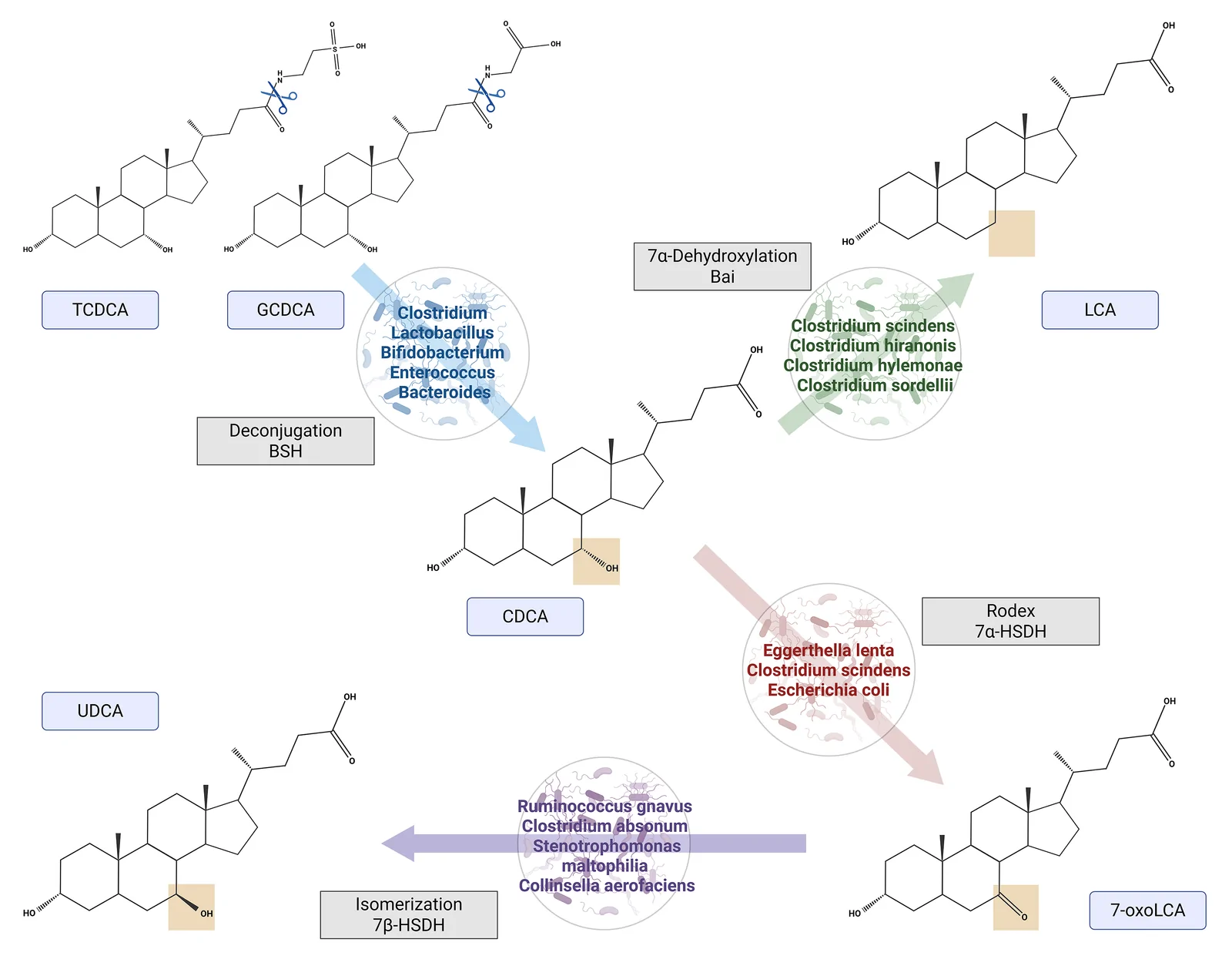
Enzymatic modifications of bile acids by the gut microbiota. Deconjugation: Catalyzed by BSH, the glycine or taurine conjugate is cleaved from the bile acid core structure. 7α-Dehydroxylation: Mediated by the bai gene cluster, the C7α-hydroxy group undergoes reductive dehydroxylation. Rodex: Catalyzed by 7α-HSDH, the C7α-hydroxy group is oxidized to a C7-oxo group. Isomerization: Catalyzed by 7β-HSDH, the C7α-hydroxy group undergoes epimerization to a C7β-hydroxy configuration. GCDCA, glycochenodeoxycholic acid; TCDCA, taurochenodeoxycholic acid; CDCA, chenodeoxycholic acid; LCA, lithocholic acid; 7-oxoLCA, 7-Oxo-lithocholic acid; UDCA, ursodeoxycholic acid; BSH, bile salt hydrolase; Bai, bile acid-inducible; 7α-HSDH, 7α-hydroxysteroid dehydrogenase; 7β-HSDH, 7β-hydroxysteroid dehydrogenase.

**Table 2:** Bacterial groups involved in bile acid transformation.

Microbial transformation pathways	Main function	Gut microbiota	Key enzymes	Substrate	Product
Deconjugation	Hydrolyzed taurine or glycine	*Clostridium*, *Lactobacillus*, *Bifidobacterium*, *Enterococcus*, *Bacteroides*	BSH	Conjugated bile acids	Unconjugated bile acids^53^, ^54^, ^55^, ^56^, ^57^
7α-Dehydroxylation	Remove the hydroxyl group at the C7 position of BAs	*C. scindens*, *C. hiranonis*, *C. hylemonae*, *C. sordellii*	Bai operon	CDCA Cholic acid	LCA DCA^58^, ^59^, ^60^
Redox and isomerization	Change the status of BA hydroxyl or ketone groups. And change the spatial configuration of the hydroxyl group (from α- to β- type).	*C.scindens*, *C.hylemonae*, *Clostridium perfringens*, *Alistipes hiranonis*, *Acinetobacter*, *Eggerthella lenta*, *Blautia producta*, *Enterorhabdus mucosicola*	3α-HSDH	DCA	3-oxoDCA^52^
		*Eggerthella lenta*, *Blautia producta*, *Ruminococcus gnavus*	3β-HSDH	3-oxoDCA	iso DCA^52^^,^^61^^,^^62^^,^^183^
		*Eggerthella lenta*, *C. scindens*, *Escherichia coli*	7α-HSDH	CDCA	7-oxoLCA
		*Ruminococcus gnavus*, *Clostridium absonum*, *Stenotrophomonas maltophilia*, *Collinsella aerofaciens*	7β-HSDH	7-oxoLCA	Ursodeoxycholic acid^52^^,^^61^^,^^62^^,^^184^
		*Eggerthella lenta*, *Enterorhabdus mucosicola*, *Clostridium scindens*, *Alistipes hiranonis*, *Clostridium hylemonae*, *Bacteroides*	12α-HSDH	DCA	12-oxoLCA^52^
		*Clostridium paraputrificum*	12β-HSDH	12-oxoLCA	epi DCA^52^^,^^61^^,^^62^

Note: This table describes the microbiota and enzymes required for the conversion of bile acids in the gut. DCA, deoxycholic acid; CDCA, chenodeoxycholic acid; LCA, lithocholic acid; BSH, bile salt hydrolase; Bai, bile acid-inducible; HSDH, hydroxysteroid dehydrogenase.

### Gut microbe–BA metabolic interactions

It is worth noting that the regulation of BA metabolism by gut microbes is colony-specific and environment-dependent. The distribution status of different microbial species and functional enzymes is dynamically regulated by factors such as host dietary structure, BAs concentration, and intestinal pH. High-fat and high-sugar diets or antibiotic interventions can disturb the homeostasis of the gut microbiota, which in turn affects the process of BA metabolism.^63^ For example, antibiotic treatment significantly reduces the abundance of 7α-dehydroxylated bacterial microbiota, leading to a decrease in secondary BA levels, disrupting BA profile homeostasis, and increasing organismal susceptibility.^64^

The disorder of BA metabolism, in turn, further aggravates the imbalance of intestinal microecology through its antibacterial effect and selective inhibitory effect, forming a bidirectional regulatory feedback loop.^65^^,^^66^ Generally speaking, the reduction of BA profile seems to be conducive to the growth of gram-negative bacteria, which can produce lipopolysaccharides (LPS), some of which have pathogenic potential. An increase in the BA profile was observed with the growth of the gram-positive Firmicutes, which promoted secondary BAs production.

In BAs-fed rats, there was a significant increase in the abundance of Firmicutes, and some microorganisms in *Erysipelatoclostridium* and *Clostridium*.^67^ In BAs-fed grass carp, feeding primary BAs tended to increase gut microbiota diversity and induce community succession, while feeding secondary BAs resulted in higher Firmicutes/Bacteroidetes ratios.^68^ Compared with normally fed mice, those administered obeticholic acid exhibited reduced endogenous BA levels and an increased Firmicutes/Bacteroidetes ratio, particularly in the small intestine, where obeticholic acid functions as an FXR agonist.^69^

Research indicates that elevated BA concentrations exert antimicrobial effects. When BA-intolerant bacteria are exposed to high BA levels, the acids dissolve phospholipids and dislodge integral membrane proteins, resulting in complete disruption of the cell membrane and consequent leakage of intracellular components.^70^^,^^71^ Beyond inducing membrane damage, BAs exert direct antimicrobial activity by disrupting gut microbial composition through multiple mechanisms: perturbing RNA secondary structures, compromising macromolecular stability, inducing DNA damage, and promoting protein misfolding.^69^

## BAs play a role in the nervous system

### BAs can cross the BBB

Existing studies have shown that BAs may enter the CNS through various pathways such as passive diffusion, active transport, and changes in BBB permeability,^72^, ^73^, ^74^ and the specific mechanism varies depending on the type, concentration of BAs and the pathological state. More than 40 kinds of primary and secondary BAs have been found in the CNS. Among the cells isolated from the brain, the ones with the highest content are CDCA, DCA, and CA. Since the enzymes required for the synthesis of secondary BAs have not been identified in the CNS, secondary BAs can only reach the CNS through the systemic circulation.^75^ Secondary UCBAs, with fewer hydroxyl groups and higher lipophilicity, may traverse the BBB via simple diffusion across its lipid bilayer, particularly at elevated local concentrations, thereby accessing the CNS. Due to their larger molecular structure and amphipathic nature, CBAs require active transport via transmembrane transporters to enter the CNS. Consequently, the abundance of CBAs in distinct CNS regions depends on the expression of solute carrier family members, including the ASBT, multidrug resistance-associated proteins (MRPs), and organic anion transporting polypeptides (OATPs). Studies confirm that CBAs serve as preferential substrates over unconjugated forms for OATP1B1 and OATP1B3.^76^ These transport proteins not only help molecules enter the bloodstream, but also protect the CNS from the accumulation of potentially toxic molecules and transport them into the blood.

### The signaling function of the interaction products between BAs and gut microbiota

BA–microbiota interactions are recognized as a crucial bridge linking gut ecology to the nervous system. Within the host, BAs undergo gut microbial biotransformation, including deconjugation, dehydroxylation, oxidation-reduction, and isomerization, generating structurally diverse secondary BAs with varied functions. These metabolites modulate neuroinflammation, immune cell activity, and neurotransmitter metabolism by activating multiple receptors, thereby playing significant roles in neurological and psychiatric disorders. The signaling functions of BAs primarily rely on their capacity to activate nuclear and membrane receptors (*e.g.*, FXR, VDR, PXR, TGR5, S1PR2). These receptors are widely distributed in intestinal epithelial cells, the liver, immune cells, and even the CNS (*e.g.*, microglia, astrocytes, neurons). Gut microbes modify BAs, transforming them into inflammation modulators.^77^ Among them, some secondary BAs generated by the metabolism of gut microbiota, such as DCA, LCA, UDCA, exhibit higher receptor affinity and specific biological effects, especially playing a significant role in regulating neuroinflammation and immune responses.

### Different BA receptors and their neuroimmune regulatory pathways

#### Farnesoid X receptor (FXR)

FXR plays a negative feedback regulatory role in neuroinflammation.^78^^,^^79^ FXR reduces the release of pro-inflammatory factors, such as interleukin-1 (IL-1), interleukin-6 (IL-6), and tumor necrosis factor-alpha (TNF-α) in microglia, astrocytes, and peripheral immune cells by inhibiting pro-inflammatory signaling pathways such as NF-κB.^80^ The activation of FXR can affect the balance of gut microbiota by regulating the composition of BAs, inhibiting pathogenic bacteria (*e.g.*, *Clostridium*), reducing intestinal inflammation and oxidative stress, reducing the intestinal leakage of endotoxins (*e.g.*, LPS), and blocking their activation of microglial cell-induced neuroinflammation through the BBB.^81^ FXR activation promotes the polarization of macrophages to the M2 anti-inflammatory phenotype, reduces the secretion of pro-inflammatory factors, and simultaneously increases the expression of the anti-inflammatory factor interleukin-10 (IL-10).^82^ Furthermore, FXR can alleviate the inflammatory response by inhibiting the nucleotide-binding oligomerization domain-like receptor protein 3 (NLRP3) inflammasome. For example, FXR-knockout mice are more susceptible to LPS-induced NLRP3 inflammasome-related sepsis, while FXR-overexpression mice are more resistant to it, indicating that BA-activated nuclear receptor FXR has an inhibitory effect on NLRP3 inflammasomes.^83^ CDCA or agonist GW4064 can inhibit the activation of the endoplasmic reticulum stress mediated protein kinase RNA-like endoplasmic reticulum kinase (PERK) by activating FXR, thereby suppressing the expression and activation of the CCAAT/enhancer-binding protein (C/EBP) homologous protein (CHOP) dependent NLRP3 inflammasome (Fig. 3).^84^

**Figure 3:**
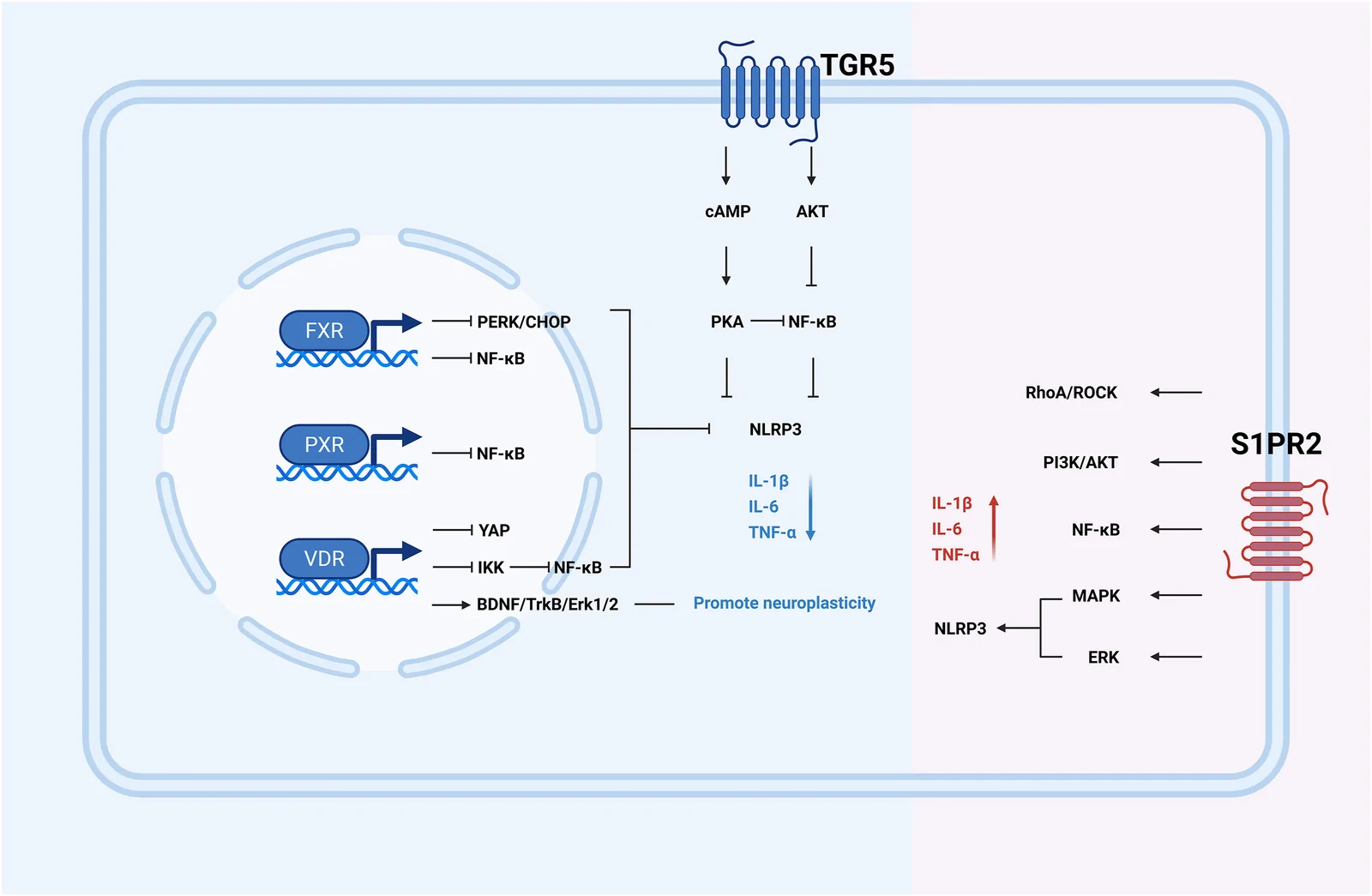
Bile acid receptor-mediated signaling pathways in neuroinflammation. Nuclear receptors FXR, PXR, and VDR, along with the membrane receptor TGR5, exert anti-inflammatory effects primarily by suppressing the NLRP3 and NF-κB pathways, thereby reducing inflammatory cytokine release. In contrast, the membrane receptor S1PR2 promotes pro-inflammatory responses. IL-1β, IL-6, and TNF-α are inflammatory cytokines. FXR, Farnesoid X receptor; PXR, Pregnane X receptor; VDR, vitamin D receptor; TGR5, Takeda G protein-coupled receptor 5; S1PR2, sphingosine-1-phosphate receptor 2.

#### Pregnane X receptor (PXR)

The activation of PXR can improve the neurodegenerative phenotype of Niemann-Pick C disease, suggesting that the activation of PXR may have a neuroprotective effect.^85^ PXR can be activated by LCA, then forms heterodimers with Retinoid X Receptor (RXR) to initiate target gene transcription, thereby participating in regulating inflammatory responses, oxidative stress, and barrier function.^37^^,^^86^ PXR activation can inhibit the NF-κB signaling pathway, reduce the pro-inflammatory factors (*e.g.*, TNF-α, IL-1β, IL-6) secreted by microglia and astrocytes, and exhibit anti-inflammatory effects.^87^ Studies have shown that PXR activation can inhibit the activation of NLRP3 inflammasome induced by oxidized low-density lipoprotein or TNF-α, thereby alleviating the inflammatory response.^88^ In addition, PXR also regulates the expression of tight junction proteins in intestinal epithelial cells, enhances intestinal barrier function, reduces the entry of enterogenic toxins (*e.g.*, LPS) into the circulatory system and the brain, and lowers the risk of neuroinflammation.^89^

#### Vitamin D receptor (VDR)

LCA modified by intestinal microbiota and its derivatives has been confirmed to act as natural agonists of VDR, activating the VDR signaling pathway. The signal transduction of VDR can activate the MEK1/2/ERK1/2 pathway. The activation of this pathway further stimulates the heterodimer formation of VDR/RXRα and recruits co-inhibitors, thereby inhibiting the transcription of the *CYP7A1* gene. In the classical pathway of BA synthesis, CYP7A1 initiates the synthesis of CA and CDCA. That is, the VDR signaling pathway may be activated by BAs and inhibit BAs synthesis, indirectly reducing oxidative stress and alleviating neuroinflammation.^90^ After activation, VDR can inhibit the pro-inflammatory NF-κB pathway by suppressing the expression of IκBα kinase (IKK) protein or promoting its ubiquitination and inactivation.^91^ Additionally, VDR activation can inhibit the NLRP3 inflammasome,^92^ or mediate the activation of yes-associated protein 1 (YAP1),^93^ which in turn suppresses NLRP3 inflammasome activation, thereby exerting anti-inflammatory effects. Furthermore, VDR signaling promotes the expression of brain-derived neurotrophic factor (BDNF), enhances neuronal plasticity and survival ability, and induces neuroprotective effects by activating the BDNF/TrkB/ERK1/2 signaling pathway.^94^ VDR can also regulate the balance of T cell subsets in the peripheral immune system, enhance the function of regulatory T cells (Tregs), and inhibit the Th1/Th17-mediated immune-inflammatory response. This mechanism may have an indirect impact on the CNS through the gut–brain axis.^95^^,^^96^

#### Takeda G protein-coupled receptor 5 (TGR5)

Whether in a conjugated state or not, BAs can act as ligands of TGR5.^97^ TGR5 can promote the formation and secretion of cAMP mediated by adenylate cyclase (AC) by coupling with stimulatory Gs protein.^98^ Relevant experiments show that the TGR5/cAMP/PKA axis can mediate the phosphorylation and ubiquitination of the NLRP3 inflammasome and inhibit the activation of the NLRP3 inflammasome.^99^ Secondly, activating the TGR5/cAMP signaling pathway with BAs or other agonists can also inhibit the transcriptional activity of NF-κB and the expression of NLRP3 inflammasome-related proteins,^100^ thereby reducing neuroinflammation and preventing microglial activation.^101^ Tauroursodeoxycholic acid (TUDCA) demonstrates neuroprotective potential in various neurological diseases, exerting neuroprotective effects by reducing neuroinflammation, apoptosis, oxidation, or endoplasmic reticulum stress.^102^ TCDCA is another CBA. It alleviates the excessive activation of astrocytes and has anti-inflammatory and immunomodulatory properties. Its mechanism of action is related to the TGR5/AKT/NF-κB signal transduction axis.^103^

#### Sphingosine-1-phosphate receptor 2 (S1PR2)

Unlike the above receptors, S1PR2 may have a pro-inflammatory effect. CBAs such as those bound to taurine and glycine are high-affinity ligands of S1PR2. S1PR2 leads to an increase in the permeability of the BBB by disrupting tight junctions.^104^ In addition, S1PR2 participates in the inflammatory response by activating classic inflammatory signaling pathways such as RhoA/ROCK,^105^ PI3K/AKT,^106^^,^^107^ and NF-κB,^108^ inducing M1 polarization in macrophages and promoting the release of inflammatory cytokines such as IL-1β and TNF-α. The activation of S1PR2 by S1P can promote the expression and activation of the NLRP3 inflammasome through the mitogen-activated protein kinase (MAPK) signaling pathway.^109^ In addition, the activation of S1PR2 by DCA can stimulate extracellular protein kinases and promote the release of lysosomal cathepsin B, thereby activating the NLRP3 inflammasome and intensifying the inflammatory response.^110^

## Gut microbiota–BA dysregulation in neuropsychiatric disorders

### Depression

In recent years, more and more evidence has shown that abnormal gut microbiota and BA metabolism play an important role in the pathogenesis of depression.^111^, ^112^, ^113^ Based on the combined analysis of clinical metabolomics and metagenomics, it was found that patients with depression presented characteristic BA metabolism disorders: the level of primary BAs was significantly increased, while the level of secondary BAs was decreased. This metabolic imbalance is closely associated with aberrant gut microbiota structure. Metagenomic sequencing reveals decreased abundance of commensal bacteria with BA hydrolase activity (*e.g.*, Lachnospiraceae, Ruminococcaceae) in patient guts, alongside increased relative abundance of *Escherichia coli* possessing dehydroxylation capacity. Consequently, the activity of key BA metabolic enzymes (*e.g.*, BSH and 7α-dehydroxylases) becomes dysregulated.^113^

It is notable that there is a significant correlation between this microbiota-metabolic disorder and cognitive impairment. Clinical studies have shown that the incidence of depression in patients with cholestatic liver disease is higher than that in the general population, and the mechanism may involve multiple pathological effects caused by excessive accumulation of BAs.^114^ The accumulation of BAs can lead to the disruption of the intestinal barrier, allowing various pathogens, endotoxins, and inflammatory factors (*e.g.*, LPS) to enter the bloodstream, triggering a strong immune response, which is manifested as a systemic inflammatory response.^73^ LPS enters the brain through the blood circulation, inducing an increase in the permeability of the BBB.^74^ Furthermore, systemic inflammation may indirectly affect the function of the BBB through the diffusion of inflammatory cytokines and further amplify the neuroinflammatory response by activating microglia in the brain, thereby exacerbating depressive symptoms. Excessive accumulation of BAs can also regulate related neurobiological processes by activating the FXR and TGR5 receptors.

Regarding therapeutic mechanisms, animal studies demonstrate that *Ginkgo biloba* extract modulates gut microbiota composition and BA metabolism. These changes alter BA profiles and consequent signaling, ultimately regulating mood and behavior in mice.^115^ Separately, metformin ameliorates depression-like behaviors in chronically stressed mice by remodeling the gut microbiota.^116^

### Schizophrenia (SZ)

The etiology of SZ has not been fully clarified yet, but both genetic and environmental factors (such as the gut microbiota) are involved in its pathogenesis. Previous studies have found that the gut microbiota structure, abundance, and microbiota-derived metabolites of patients with SZ are abnormal, and this dysregulatory state is significantly associated with the down-regulation of BA synthesis and the up-regulation of lipid metabolism.^117^^,^^118^ The BA profile of patients with SZ shows an obvious disorder. The reduction of BAs such as CA and CDCA, which play a neuroprotective role, may cause a decline in the cognitive level of patients with SZ.^119^ Because the reduction amplitude of CDCA level is greater than that of CA level, the ratio of CA to CDCA in patients with SZ is higher. Studies have found that the CDCA level in patients with SZ is significantly reduced,^118^ and CDCA treatment has been proven to improve the mental symptoms of patients with cerebrotendinous xanthomatosis (CTX).^120^ A recent case report indicates that UDCA supplements have shown clinical efficacy and safety for refractory SZ.^121^ It indicates that UDCA may be a feasible therapeutic target for personalized SZ treatment. Patients with SZ exhibit disruption of the gut virome-bacteriome network. Intestinal viruses (predominantly bacteriophages) modulate gut microbiota structure and metabolic functions through predator-prey dynamics, thereby influencing BA synthesis. This cascade induces immune dysregulation and inflammatory responses, potentially correlating with the host's cognitive impairment.^122^

### Alzheimer's disease (AD)

Intestinal microbiota dysregulation may be related to AD,^123^ mainly interacting bidirectionally with the CNS by generating intestinal microbiota metabolites that affect neuroimmunity.^124^^,^^125^ The metabolites of the intestinal microbiota can be directly derived from bacteria, or from diet, or from the metabolic transformation of substrates from the host.^126^ The currently known metabolites of the intestinal microbiota mainly include short-chain fatty acids, BAs, choline metabolites, phenols, indole derivatives, vitamins, and lipids, *etc*. These metabolites mainly play a role in regulating lipid metabolism and glucose homeostasis in the body. Recent studies have found that the changes in metabolites caused by gut microbiota imbalance may further affect the pathology of AD through pathways such as triggering neuroinflammation and apoptosis of nerve cells.^127^, ^128^, ^129^

UDCA and TUDCA can inhibit apoptosis by stabilizing the mitochondrial membrane.^130^ It has been found that UDCA can also affect the genes regulated by regulating NF-κB inhibitory protein α and NF-κB, thereby protecting the BV-2 microglia cell line from the toxic hazards caused by the accumulation of β-amyloid (Aβ).^131^ In addition, TUDCA has been proven to have neuroprotective effects. It can prevent the occurrence of Aβ -induced mitochondrial apoptotic pathways by inhibiting the production of oxygen free radicals, reducing endoplasmic reticulum stress, and stabilizing unfolded protein responses.^132^ Meanwhile, TUDCA can also reduce the activation of glial cells, thereby lowering the expression of pro-inflammatory cytokines such as TNF-α. In addition, it can reduce synaptic loss and neuronal death, thereby improving the pathological state of AD.^133^^,^^134^ However, due to the changes in the gut microbiota of AD patients, the metabolism of BAs is altered. The disruption of BA biosynthesis will further lead to alterations in cholesterol metabolism, giving rise to an increased production of cytotoxic BAs.^135^ Current studies have found that compared with normal people, the ratio of secondary BAs to primary BAs in the serum of AD patients, such as the ratio of DCA to CA, is significantly increased. Moreover, this change is significantly associated with reduced cognitive ability, and this alteration is also related to the accumulation of Aβ, total tau protein, and phosphorylated tau protein.^136^ Among them, UCBA, glycolithocholic acid, taurolithocholic acid, taurodeoxycholic acid, *etc*., are cytotoxic BAs. They can cause mitochondrial dysfunction by destroying the mitochondrial membrane, reducing the mitochondrial membrane potential, and promoting the production of reactive oxygen species, leading to an increase in inflammation and apoptosis. All these may cause damage to nerve cells in the brain, thereby aggravating AD. In addition, BAs can also act as signaling molecules and affect the activation of some nuclear receptors, membrane receptors, and neurotransmitter receptors such as FXR, TGR5, PXR, and N-methyl-d-aspartate (NMDA) receptors. Previous studies have found that CDCA, LCA, and DCA can inhibit the activation of NMDA receptors. The appropriate activation of NMDA receptors is related to the functions of learning and memory. Under pathological conditions, the disorder of these BA levels may lead to the dysfunction of NMDA receptors, thereby causing learning and memory disorders.^137^ BA-activated PXR may promote the clearance of Aβ protein by up-regulating P-glycoprotein, which has potential therapeutic value for AD.^138^ BAs can also regulate glucose and lipid metabolism by activating FXR and TGR5 receptors. Disorders of these two metabolisms may lead to diabetes and cerebrovascular dysfunction, further increasing the possibility of AD onset.^139^

### Parkinson's disease (PD)

PD is usually the result of the combined effect of multiple factors. Genetic susceptibility, exposure to environmental toxins, and aging of the nervous system interact with each other. Through complex mechanisms such as oxidative stress and mitochondrial dysfunction, a large number of dopaminergic neurons in the substantia nigra undergo degeneration and loss, eventually leading to PD. The Lewy body formed by the abnormal accumulation of α-synuclein (α-Syn) is the core factor of the disease.

BA production may play an unrecognized role in PD pathogenesis, with gut microbiota alterations directly modulating BA synthesis. PD patients exhibit significantly reduced gut microbial diversity and depleted beneficial bacteria, alongside abnormal expansions of Proteobacteria (*e.g.*, *Escherichia coli*) and Firmicutes. These dysbiotic changes perturb BA metabolism, elevating levels of secondary BAs (*e.g.*, DCA and LCA), which demonstrate neurotoxicity by impairing mitochondrial function, inducing oxidative stress, and triggering neuroinflammation, thereby exacerbating dopaminergic neuron degeneration. Critically, this dysmetabolic signature is unique to PD patients and absent in healthy controls, positioning BAs as detectable biomarkers for early diagnosis and tracking of disease progression.^140^ The latest theory suggests that the abnormal aggregation of α-Syn may start in the intestine and travel up to the brain via the gut–brain axis. Functional amyloid proteins produced by the intestinal microbiota can promote the cross-aggregation of α-Syn, while secondary BAs may accelerate this process by disrupting the intestinal barrier.^141^ In addition, BAs affect neuroprotection and inflammatory regulation by activating the nuclear receptor FXR or inhibiting the TGR5 signaling pathway.

In PD, the c-Jun N-terminal kinase (JNK) signaling pathway is involved. As a key member of the MAPK family, JNK can be activated by various cellular stressors such as oxidative stress and inflammatory cytokines. Previous studies have suggested that inhibiting JNK has a protective effect on the apoptosis of dopaminergic neurons. During the pathogenesis of PD, the JNK pathway constitutes a central stress activation response, and an elevated level has been found in the PD brain.^142^^,^^143^ UDCA and TUDCA have been studied and found to play the role of neuroprotective agents in PD by preventing apoptosis and regulating JNK activity and cellular redox threshold.^144^ Rotenone can cause various changes in mitochondria related to PD, inducing the generation of free radicals, inhibition of complex I, and apoptosis. TUDCA treatment has a protective effect on the toxicity induced by rotenone.^145^ The neuroprotective effect of TUDCA on MPTP toxicity was observed in a study conducted on mice lacking glutathione S-transferase pi (an endogenous JNK inhibitor).^144^ 1-Methyl-4-phenyl-1,2,3,6-tetrahydropyridine (MPTP) is a neurotoxin that induces PD in animals. TUDCA pretreatment significantly reduced the exhaustion of dopaminergic neurons and the loss of dopaminergic fibers caused by MPTP. TUDCA prevents MPTP-induced neurodegeneration by delaying and reducing the loss of dopaminergic cells.^146^ Administration of TUDCA also has a neuroprotective effect on substantia nigra dopaminergic neurons transplanted into PD rodent models.

### Amyotrophic lateral sclerosis

Amyotrophic lateral sclerosis is a rare progressive neurodegenerative disease. Its core feature is the irreversible death of motor neurons, leading to muscle atrophy, weakness, paralysis, and ultimately affecting breathing and swallowing functions, resulting in the patient's death within 2–5 years.^147^

Patients with amyotrophic lateral sclerosis generally have abnormal manifestations of intestinal function, which are closely related to the imbalance of gut microbiota, and the imbalance of microbiota will further interfere with the production of BAs. A case–control study indicated that the levels of serum UDCA, TUDCA, and GUDCA in patients with amyotrophic lateral sclerosis showed an increasing trend.^148^ TUDCA has been found to show benefits in slowing down the progression of amyotrophic lateral sclerosis.^149^ In September 2022, the U.S. Food and Drug Administration approved the combination of sodium phenylbutyrate and taurursodiol as a potential therapeutic candidate for amyotrophic lateral sclerosis. This decision followed prior findings that the combination extended patient survival by 10 months in clinical trials (Fig. 4).^150^^,^^151^

**Figure 4:**
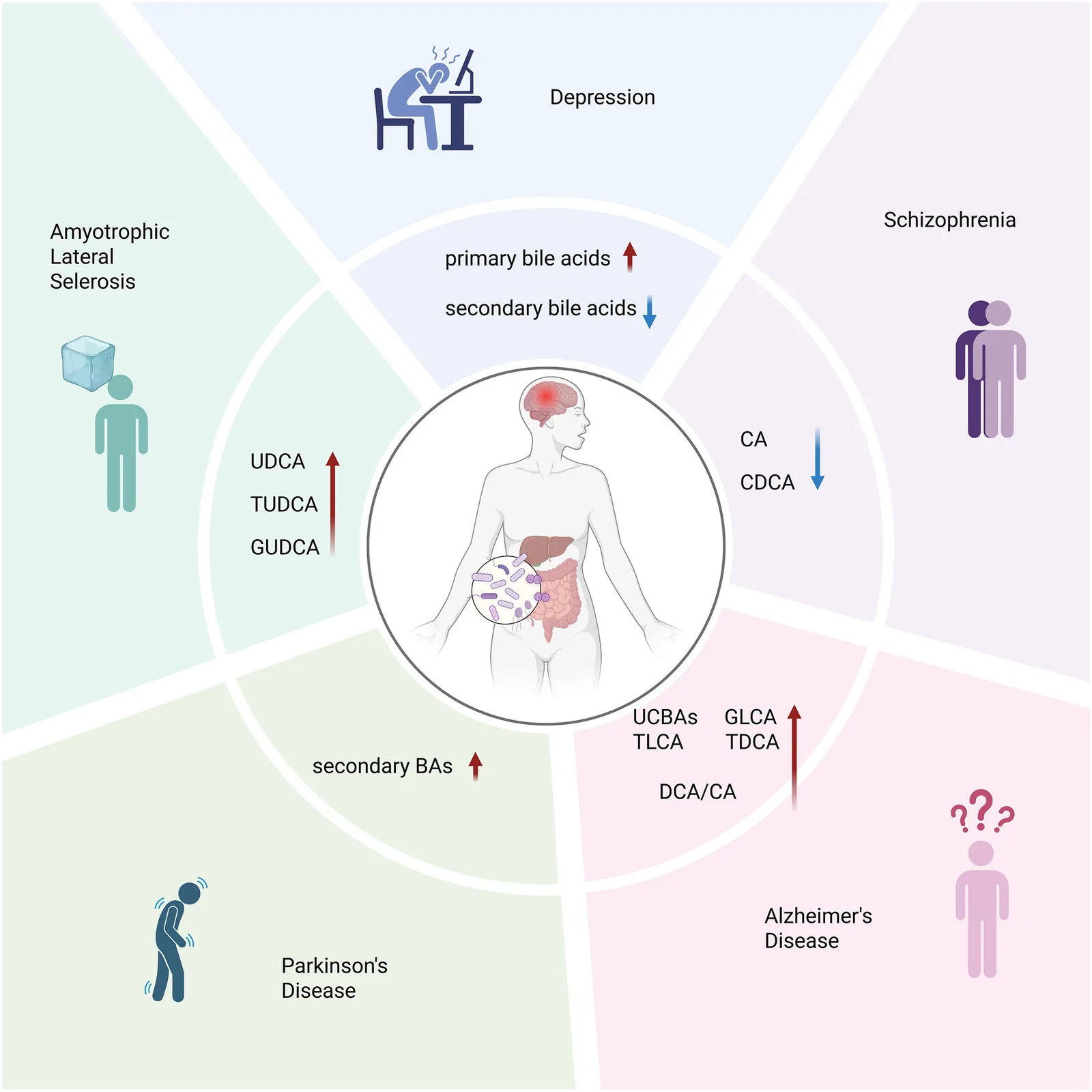
Disrupted BA metabolism contributes to the pathogenesis of various neurological disorders. BA, bile acid; CA, cholic acid; CDCA, chenodeoxycholic acid; UDCA, ursodeoxycholic acid; TUDCA, tauroursodeoxycholic acid; GUDCA, glycoursodeoxycholic acid; UCBAs, unconjugated bile acids; GLCA, glycolithocholic acid; TLCA, taurocholic acid; TDCA, taurodeoxycholic acid.

### Limitations of clinical evidence and translation barriers

Although the microbiota–BA–brain axis demonstrates significant therapeutic potential for neurological diseases, current research evidence still has notable limitations, and its translation to the clinic faces multiple challenges.

Existing clinical evidence has numerous limitations. In terms of study scale and design, most human studies have limited sample sizes, with issues such as small cohorts (*n* < 100), inclusion of only male volunteers, and exclusion of obese volunteers. This results in insufficient statistical power, potentially making it difficult to detect small but biologically significant effects.^152^, ^153^, ^154^, ^155^ Due to the inherent limitations of clinical research, current studies primarily originate from cross-sectional designs. The vast majority find significant differences in BA profiles between patients with psychiatric/neurological disorders (*e.g.*, depression, AD) and healthy controls. While these cross-sectional studies reveal important associations, they cannot clarify the direction of causality. Consequently, we cannot determine whether abnormal BA metabolism is a driver of the disease or merely a consequence of the disease state.^156^ Regarding detection methods and sample sources, variations in BA detection methodologies and sample origins make it difficult to directly compare results across studies. BA samples are derived from diverse sources, including serum, plasma, feces, or intestinal content. Currently, most studies measure BA levels in both serum and feces, which helps mitigate discrepancies when comparing studies using different matrices.^157^^,^^158^ However, the mass spectrometry methods employed in different studies may vary in their accuracy for quantitative analysis, leading to incomplete assessment of some low-abundance but highly active secondary BAs. Furthermore, plasma BA levels are susceptible to transient influences like food intake and circadian rhythms, whereas fecal BAs reflect the overall metabolic profile. The relationship between these measures and both the local gut microenvironment and CNS effects remains to be elucidated.^159^, ^160^, ^161^, ^162^ Furthermore, regarding microbiome heterogeneity and confounding factors, the gut microbiota exhibits substantial inter-individual variation and is profoundly influenced by confounders such as dietary patterns, medication use, and comorbidities. Failure to adequately adjust for these factors statistically can severely distort the true assessment of the microbiota-BA-disease relationship.^163^, ^164^, ^165^, ^166^, ^167^

Therapeutic strategies based on this axis face severe challenges when moving towards clinical translation. Drug delivery efficiency is a primary obstacle. The BBB can block over 98% of small-molecule drugs and almost all large-molecule therapeutics from entering the brain, limiting central targeting efficacy.^168^ Simultaneously, the oral administration route presents specific barriers for different therapeutic formulations: live biotherapeutic products (*e.g.*, probiotics) primarily face challenges of survival and colonization in the harsh gastrointestinal environment,^169^, ^170^, ^171^ whereas bioactive molecules (*e.g.*, BAs and their analogs) mainly confront challenges of premature metabolism, degradation, and low bioavailability.^172^, ^173^, ^174^, ^175^ BAs can simultaneously activate multiple receptors, and the expression and function of these receptors vary across organs and cell types. Therefore, developing ligands with tissue and receptor specificity is crucial to avoid unpredictable systemic side effects caused by off-target effects.^176^ Engineering microbes—modifying commensal bacteria via synthetic biology to equip them with the ability to sense disease signals and precisely produce therapeutic substances (*e.g.*, BAs or neurotransmitters)—offers a novel approach to addressing targeted delivery challenges.^177^^,^^178^ However, their long-term safety requires evaluation, and the potential for modulating BA metabolism to disrupt host metabolic homeostasis is a core issue that must be resolved before translation.^179^^,^^180^ Additionally, in biotherapeutics, patient heterogeneity determines treatment efficacy. For instance, different baseline microenvironments may completely hinder the colonization and function of exogenous strains. Factors such as host genotype, dietary habits, and medication history introduce potential unknown risks into treatments, posing challenges for clinical trial design and interpretation of results.

In response to the clinical translational challenges mentioned above, future strategies should focus on developing advanced delivery systems—such as acid-resistant encapsulated nanocarriers—to deliver probiotics or metabolites with improved bioavailability and targeting capability. Personalized therapeutic plans should be designed based on patients' microbiome profiles, genetic backgrounds, and lifestyle habits. At the same time, long-term safety evaluations and follow-up studies are essential. In summary, although therapies based on the gut–brain axis hold great potential, it is crucial to clearly recognize and address these translational challenges.

## Conclusions

In conclusion, the functional scope of BAs has expanded beyond traditional roles in lipid digestion, evolving into pivotal signaling hubs that interconnect the gut microbiota, neuroimmune system, and CNS. Gut microorganisms biotransform primary BAs into secondary species via deconjugation and dehydroxylation. These metabolites are further converted into microbe-conjugated bile acids under catalysis by BSH, thereby remodeling microbial community structures and modulating host physiology. By activating BA-specific receptor networks (*e.g.*, FXR, TGR5), these compounds directly regulate neuroimmune homeostasis. Notably, dysregulated BA-microbiota crosstalk compromises intestinal epithelial barrier integrity, triggering systemic low-grade inflammation. This process exacerbates central neuroinflammation by altering BBB permeability, ultimately contributing to the pathogenesis of neurodegenerative disorders (*e.g.*, AD, PD) and depression. This dysregulation is therefore established as a critical pathophysiological basis for these conditions.

Recent breakthrough research reveals that BAs orchestrate immune responses, suppress neuroinflammation, and maintain gut–brain axis homeostasis through synergistic actions of multiple receptors (*e.g.*, FXR, TGR5), thereby safeguarding neural functional integrity. However, three critical knowledge gaps persist: First, the mechanisms of BA receptors have not yet been fully elucidated. Current research mainly focuses on FXR and TGR5, while the roles of other receptors in neuroimmunity remain insufficiently explored. Second, functional heterogeneity among distinct BA species in disease contexts lacks systematic investigation. Third, the complexity of multidimensional microbiota-BA-neural interactions poses significant challenges.

To specifically address these knowledge gaps, future research must follow a clear, prioritized pathway. The primary task is to establish standardized methods for BA detection and microbiome analysis, and to initiate large-scale longitudinal cohort studies. This initiative aims to obtain high-quality, comparable data to systematically reveal the dynamic changes of different BA molecules and their temporal sequence in relation to disease risk. Based on acquiring reliable data, focus should shift to in-depth mechanistic studies, investigating the roles of incompletely elucidated BA receptors in neuroimmune functions and uncovering the molecular basis for the functional heterogeneity of BAs. Ultimately, translate these basic research findings into clinical applications. This includes developing receptor-selective ligands to precisely modulate specific pathways, designing controllable engineered microbial systems to decipher and intervene in complex multi-dimensional interaction networks, and subsequently conducting biomarker-based early-stage clinical trials. The ultimate goal is to provide a new precision paradigm for the prevention and treatment of neuroimmune diseases.

## CRediT authorship contribution statement

**Xiaoqi Pei:** Writing – original draft, Conceptualization. **Xiaoli Wang:** Writing – original draft, Investigation. **Hongji Lu:** Writing – original draft, Resources. **Jialin Wu:** Resources, Investigation. **Lu Xu:** Resources, Investigation. **Xiaoyin Chen:** Writing – review & editing, Supervision. **Li Deng:** Writing – review & editing, Resources, Supervision. **Huachong Xu:** Writing – review & editing, Resources, Project administration, Supervision.

## Funding

This study was financially supported by the 10.13039/501100001809National Natural Science Foundation of China (No. 82474207, 82305020), the 10.13039/501100021171Guangdong Basic and Applied Basic Research Foundation of China (No. 2024A1515011809, 2024A1515011014, 2025B1515020040, 2024A1515030171), the Guangdong Provincial Key Laboratory of Traditional Chinese Medicine Informatization (China) (No. 2021B1212040007), and the Youth Science and Technology Talent Support Project of Jinan University Science and Technology Association (Guangzhou, China) (No. 21624222). The author gratefully acknowledges the support of the K.C. Wong Education Foundation.

## Conflict of interests

The authors have no competing interests to declare.
